# The Problem of Expertise in Knowledge Societies

**DOI:** 10.1007/s11024-016-9308-7

**Published:** 2016-09-27

**Authors:** Reiner Grundmann

**Affiliations:** School of Sociology and Social Policy, University of Nottingham, University Park, Nottingham, NG7 2RD UK

**Keywords:** Experts, Expertise, Science, Knowledge, Knowledge societies, Decision-making, STS

## Abstract

This paper puts forward a theoretical framework for the analysis of expertise and experts in contemporary societies. It argues that while prevailing approaches have come to see expertise in various forms and functions, they tend to neglect the broader historical and societal context, and importantly the relational aspect of expertise. This will be discussed with regard to influential theoretical frameworks, such as laboratory studies, regulatory science, lay expertise, post-normal science, and honest brokers. An alternative framework of expertise is introduced, showing the limitations of existing frameworks and emphasizing one crucial element of all expertise, which is their role in guiding action.

## Introduction

The question what expertise is and what experts do, what role they play in society and what role they should play is a recurrent theme not only in the recent literature. Authors argue about the desirability of recognition of specific forms of knowledge and expertise and their proper place in the decision-making process in modern societies. Many debates have revolved around the question if experts should have a privileged position in democracies. Such questions take for granted that we know what experts are and in which social context they operate. The argument put forward here claims that we need to go back to basics. We need to understand expertise more deeply and contextualize it in modern society and the sociological literature. I will develop a framework that allows us to better understand how expertise emerges and how it works in society, what its main characteristics are, and how it relates to common notions of scientific and professional expertise.

The paper has the following structure. I will first provide a definition of expertise, which I link to a sociological account of what has been called the rise of the knowledge society. My effort is indebted to a line of thought that includes authors such as Daniel Bell, Peter Drucker, and Nico Stehr. I will then show, by way of a critical review, how several influential frameworks from the field of Science and Technology Studies (STS), broadly conceived, have conceptualised expertise. The limitations of these approaches will be discussed and confronted with a relational concept of expertise.

## What are Experts and What do They do?

Definitions of expertise usually revolve around the specialist craft or knowledge a person is said to possess. An often-used synonym is specialist. The opposite of an expert or specialist is an amateur, or a layperson (the term lay expert in this sense is an oxymoron). These characteristics of being a specialist apply to several roles in modern society, especially the professional and the scientist. Sometimes experts are thought to have moral virtue, besides their technical competence. This virtue can be captured by terms such as disinterestedness or impartiality^1^ (Collins 2014a,b; Laski 1930; Nelkin 1975; Shapin 2008; Turner 2001).

From this short overview the following list of issues can be derived:

(1) There is a fundamental difference between experts and non-experts; (2) experts are located in the professions and in science; (3) experts possess technical skills, including manual and intellectual skills; (4) experts are impartial which makes their advice trustworthy. Within the remit of this paper not all issues can be discussed in the same depth. I will focus on the knowledge dimension of expertise and exclude *manual* skills from the discussion. And I will comment on the issue of impartiality only in passing. What is missing from the above list are two crucial *relational* aspects. The list above treats expertise as something that persons have, a body of knowledge that can be attributed. However, expertise is essentially something delivered at the request of someone else who wants it. This makes expertise relational in a double sense: it relates to clients and it relates to their needs, which often is the need for guidance in decision-making.^2^ It is the aim of this paper to develop and explore this double relationship.

This relational concept can be summarized as follows: experts mediate between the production of knowledge and its application; they define and interpret situations; and they set priorities for action. Experts are primarily judged by clients, not necessarily by peers (professional or scientific); and they rely on trust by their clients (cf. Stehr and Grundmann 2011; see Martin 1973 for an early exposition). As Martin (1973: 159) aptly remarked, ‘[e]xpertness is an ascribed quality, a badge, which cannot be manufactured and affected by an expert himself, but rather only can be received from another, a client’. Aristotle shrewdly remarked that ‘the guest will judge better of a feast than the cook’.

Conventional examples of experts are science advisory bodies and consultants to decision-makers, including legal counsellors and medical doctors. However, there is an emerging form of expertise which becomes ever more important. This has been described as lay expertise, and it has been thematized in the literature. Examples are citizens being involved in risk decisions about health and the environment, but also the growing number of Internet platforms on which citizens give advice based on their own knowledge and experience, on matters ranging from finance to cultural entertainment. Much of the academic literature on expertise implicitly or explicitly wants to say something about the quality or desirability of specific forms of expertise, often using scientific expertise as yardstick or model. This tends to obscure vitally important aspects of expertise.

Peter Dear (2004) gives a definition of expert which emphasizes the importance of personal experience and tacit knowledge. It also draws attention to the fact that an expert is defined by others as an expert:

‘An expert . . . is someone who is reckoned to be likely to be experienced in the relevant matters. … In effect, shared experience relies on the ability to recognize a kind of attribute or property that people (“experts”) can be said to possess. Expertise thus resembles “tacit knowledge” as understood by scholars in science studies’ (Dear 2004: 206–207).

Many people may advertise themselves as experts but they will only be able to play this role when a client starts to use their service (see Martin 1973). The expert has thus to be trusted by a client—in contrast to a professional who is certified by a professional organization (see the traditional definition by Abbott 1988; recent scholarship sees challenges to the status of professions, e.g. Evetts 2011).

The word *expert* has its root in the Latin verb *experiri,* to try. An *expertus* is someone who is experienced, has risked and endured something, is proven and tested. Closely related is the word *experimentum*. The semantic field opened up through this etymology has led to an emphasis on tacit knowledge and experiments, which some STS scholars have emphasized as crucial component of scientific work. This definition is restricting the meaning of expertise to the aspect of people in possession of some kind of knowledge or skills.

But experts are not only characterised by their embodiment of skills and experience. What matters is their performance. They are asked to share their knowledge (or at least some of it) and advise others what to do. In this way knowledge becomes a capacity to act (Stehr 1994). The Latin verb *consulere* means to ask, to question or to examine; to give advice. And the verb *consultare* is used as a synonym for *curare* – to take care of someone, or to worry about someone. There are well known social functions where professional roles refer to the term consultant–for example, specialist doctors, consultants advising business firms, or advisers of politicians. It is true that consultants draw on specialised knowledge, but this knowledge need not be a comprehensive reflection of the current state of the relevant science. Personal experience, judgement, and trustworthiness are much more relevant. Because consultants are judged (and sometimes paid) by their clients, past achievements, reliability and credibility are of vital importance. Using the term expert as a synonym for consultant emphasises these aspects of the role.

Expertise thus defined contrasts not only with the notion that people possess something (knowledge or skills), it also contrasts with the notion that expertise is primarily linked to scientific activity. Of course, scientists may become experts in that they advise clients. But often their advisory activity is only loosely related to their core expertise, and clients request their advice because it bestows prestige and legitimation.^3^ The scientists’ main activity is generating new scientific knowledge, not necessarily creating advice for action or guiding decision-making. To put it in a nutshell, there can be knowledge, and scientific knowledge, without expertise (see Sassower 1993).

Classical sociologists were well aware of the difference between knowledge and action. Marx (1867) proclaimed that in the beginning there was a decision: ‘In their difficulties our commodity owners think like Faust: “Im Anfang war die Tat.” [“In the beginning was the deed.” – Goethe, *Faust*]’. They therefore acted and transacted before they performed research. Durkheim said ‘Life cannot wait’ (Durkheim 1915: 431), and scientific knowledge is its opposite, characterized by limited or even absent opportunities to take action. According to Durkheim, beliefs provide the impetus for action, whereas science, ‘however far one might want to pursue it . . . always keeps its distance from the action’.

Likewise, Simmel (1890: 1) thought that it is more important to a human being to do something than to know how he managed to do it, and the insight into how the action was accomplished comes afterward. Society cannot wait until its problems are solved scientifically; it must decide which course of action to take. Scientific knowledge operates under conditions that allow waiting, gaining of distance and overview. The absence of urgency in scientific knowledge production makes science seem inappropriate for situations in which urgency becomes the most important characteristic. If we were waiting for conclusive evidence before taking action, decision-making would be paralysed. Knowledge for action can only ever be ‘good enough’, a judgement made by decision-makers with the help of experts (with experts defined here and in the following as consultants and advisors, not research scientists, although the latter are often performing such tasks). This pragmatist take on knowledge and decision-making sums up the proposed framework for the analysis of expertise.

## Traditional, Industrial, and Knowledge Societies

Turning to the historical and social context of expertise, let me start with an observation by Zygmunt Bauman. In his book *Legislators and Interpreters*, Bauman (1987) writes about the role of the intellectual and introduces the terms ‘legislators’ and ‘interpreters’ in order to illustrate the difference between modern and post-modern societies. In modern societies, Bauman suggests, the role of intellectual is akin to the legislator, whereas in post-modern societies intellectuals are more like interpreters (see also Osborne 2004). Similar to other theorists of postmodernity Bauman points to the fact that different social forms lead to the emergence of different forms and constellations of power and knowledge, and that post-modern societies deny intellectuals the role of all-knowing, guiding and leading figures.

This is a useful sociological point of departure but needs to be more nuanced. Instead of talking about modern and post-modern societies one should distinguish between traditional, industrial and knowledge societies. One should also allow for the possibility that former traits will continue in later stages of societal change. This would mean that knowledge societies are likely to have elements of both traditional and industrial societies. Rather than positing a rigid separation of forms of societies and types of experts one should assume a mixture of them.

With regard to expertise this means that in a traditional society decisions are justified by references to God, Nature, or the good life (Douglas 1966). Industrial societies make references to wealth and health more prominent so that efficiency, competition, inclusion and concerns about the consequences of the industrial mode of production and its impacts on humans and the natural environment become important (Beck 1992). In this society science appears as an arbiter in many contentious issues, as Bauman’s role of ‘legislator’ indicates. Technocracy is the name of the game.

As we move into the knowledge society religious or moral references have not completely disappeared as one can observe in many debates, and arguments continue about the leading role of science, allegedly providing some ‘rational’ guidance for society. But like Bauman’s intellectuals who have lost their role as legislators, science has lost its position of authority. Partly this was due to disillusionment with scientific and technological projects after the Second World War, but also due to the emergence of a multiplicity of knowledge sources (Jensen, Lahn, and Nerland 2012; Stehr and Grundmann 2011).^4^

Not only have knowledge sources proliferated, appeals to scientific expertise and authority have become more problematic as a result of technical and environmental disasters which have been blamed on a failure of scientific guidance or foresight. As Camille Limoges pointed out,

‘There was a time when the mobilization of experts was a taken-for-granted, unproblematic aspect of decision-making processes. Experts would ‘educate the public’ and, in so doing, prevent the eruption of controversies. It is upon such a basis that technology assessment, *qua* expert knowledge, was first established. Confidence in the power of expertise has now vanished’ (Limoges 1993: 417).

Meanwhile, suspicion about scientists, engineers, politicians, and business corporations who use science in order to push an agenda, or to promote or undermine a technology has become widespread. Dorothy Nelkin (1975: 36) said as much in the mid-1970s: ‘[E]xperts are resented and feared. While the reliance on experts is growing, we see a revival of Jacksonian hostility toward expertise, and of the belief that common sense is an adequate substitute for technical knowledge’ (see also Beck 1992; Collins 2014a,b; Goldacre 2012; Mirowski 2011; Wood and Mador 2013).^5^

However, suspicion about elite expertise is not the only feature of modern societies. There is plenty of evidence that social change over the past decades has made societies more knowledge dependent. New products embody higher inputs of knowledge. Rising proportions of the population everywhere are university graduates (Frank and Meyer 2007), a trend already diagnosed by Daniel Bell (1973) and Peter Drucker (1968).

The pool of knowledgeable citizens has increased enormously between the 1960s and today. Many more people are highly educated and earn their money as ‘knowledge workers’ (Brint 2001). At the same time, as traditional ties have been loosened, people cannot rely on received wisdom and traditional ways of life. They have to make decisions about their lives themselves. This trend towards individualisation and risk decisions has been well described by Beck (1992), Giddens (1991) and others. This means that individuals are seeking expertise, and may find it being offered by non-certified experts. The trend is powerful and has not spared the professions, either. They have been challenged by the growing number of citizens some of whom have university degrees and are knowledgeable and interested in issues that were left to professionals and scientists only a few decades ago. As Jensen et al. (2012: 2) put it, ‘expert knowledge is generally contested and branded with uncertainty. This is partly related to the emergence of the information society, which paves the way for extensive distribution of knowledge and information that is open to all without necessarily having gained the endorsement of professional or institutional authorities’.

In contemporary society the reliance on expertise is ubiquitous, with people being both potential providers and clients of expertise. Many social situations require expert judgement, no matter if we look at career advisors in schools, financial advisors (pensions and mortgages), or partnership and marriage advisors.^6^ A recent study on a health campaign in Finland finds that ‘field experts’ are the most visible actors in the media (Väliverronen 2014). These are nutrition therapists and personal fitness trainers who ‘did not appear so much as sources of scientific information, but instead as authorised users of that information. They gave advice, encouraged citizens to record their weight, and offered quite detailed prescriptions in their interviews of the sort of lifestyles people should lead in order to achieve their ideal weight’ (Väliverronen 2014: 525).

The argument so far describes a paradoxical development: it lays out that the emergence of the knowledge society has led to a proliferation of, and dependence on expertise. It has led to a loss of trust in scientific experts while at the same time generating forms of expertise that are not based on professional accreditation or scientific reputation (see also Weingart 1999). On the supply side there is more expertise on offer, including new forms of expertise from below. On the demand side more expertise is requested, and the range of requesting actors includes politics, firms, NGOs and citizens (Brown 2009). Experts combine knowledge with its interpretation, and most crucially with an action orientation, making abstract knowledge actionable, or advising clients on how to act in the absence of certain knowledge.^7^ These are important pointers to be remembered for the argument below.

Stephen Turner (2001) attempts to provide a typology of expertise, looking at the supply and demand side. On the supply side he gives examples ranging from economists, theologians, public administrators and social workers, and from single intellectuals to think tanks. On the demand side he identifies governments (‘high politics’), public administration (‘bureaucratic apparatus’), NGOs, business firms, and citizens. This leads to a typology of expertise, based on different modes of claiming and requesting cognitive authority. Perhaps the most important issue here is the expert’s relation to an audience and clientele. It is crucial who commissions or pays for the expertise, and how clients come to trust it. It is also appropriate to distinguish between experts that advise ‘high politics’ and those that provide advice to other social users, such as charities or general audiences.

In Table 1 I provide a simplified version of this typology, presenting supply and demand functions in different actor constellations. On the supply side, I distinguish between scientific, professional and field expertise. On the demand side, I distinguish between politics, business, NGOs and private citizens. I use the term ‘field experts’ to denote the kind of ubiquitous expertise that has been identified as important by much of the literature. The examples should help in describing some essential roles and help us identify the specific type of expert-client interaction that is examined by different approaches discussed below.

**Table 1:** Typology of expertise

Supply	Demand			
	Government	Business	NGOs	Citizens
Scientific experts	*Advisory committee*	*Consultancy*	*Member, advisor*	*Media exposure*
Professional experts	*Lawyers, engineers etc.*	*Consultancy*	*Lawyers*	*Lawyers, doctors, financial advisors*
Field experts	*Roundtables, deliberative democracy*	*Crowdsourcing*	*Members, supporters*	*Patient groups, action groups, online blogs, self-help advice*

Before I do this, I want to take a quick look at the example of field expertise in health care as opposed to legal settings, to take two old professional domains. Medical doctors are part of the medical profession but they did not necessarily produce the knowledge they are using when treating patients. They are not scientists but medical experts (in the sense of consultants—in English a specialist doctor is called appropriately *consultant*).^8^

Likewise, lawyers did not necessarily produce the knowledge they are applying when making a case. By way of contrast, the legal expertise seems to be much less challenged by field expertise. It is an open question if this is due to professional strategies of defending their specialist status or to their legally granted monopoly status. There are no comparable Internet platforms run by field experts as is the case in the medical domain, and the evidence seems to suggest that defendants without professional legal counsel fare worse in front of the courts (Sandefur 2015).

In what follows I will critically discuss five approaches from the literature in STS that have conceptualised expertise. I will show their limitations and argue for an alternative approach that takes into account the double relation of expertise, between experts and clients; and between experts and decision-making.

## Five Influential Views and Their Problems

### Laboratory Studies

Harry Collins has made a very visible contribution for the analysis of expertise in contemporary society (Collins and Evans 2002, 2007; Collins 1985, 2014a,b). He affirms the point made above about the difference in urgency within politics and science. Different time scales apply in policy decisions and scientific research: one is urgent; the other always needs more time to do further research.

Collins and Evans critically review the move in science studies towards relativizing faith in science, and attendant calls for an extension of scientific expertise. They argue that there are good reasons to give scientific expertise a special place in society and its decision-making procedures:

‘One of the most important contributions of the sociology of scientific knowledge (SSK) has been to make it much harder to make the claim: “Trust scientists because they have special access to the truth”. Our question is: “If it is no longer clear that scientists and technologists have special access to the truth, why should their advice be specially valued?” This, we think, is the pressing intellectual problem of the age’ (Collins and Evans 2002: 236).

Collins and Evans pose a related question about the scope of non-scientists’ participation in decision-making: ‘How far should participation in technical decision-making extend?’ Science studies have shown that there is ‘more to scientific and technical expertise than is encompassed in the work of formally accredited scientists and technologists, but it has not told us how much more’ (Collins and Evans 2002: 237).

Two important issues are to note here. One is about participation in decision-making, the other about participation in knowledge creation. Collins and Evans’s term ‘socio-technical decision-making’ seems to relate to both. However, the authors do not elaborate on the potential ambiguity of this term. Instead, they introduce another distinction, between interactional and contributory expertise. The first is defined as ‘enough expertise to interact interestingly with participants’, the second as ‘enough expertise to contribute to the science’. According to Collins (Collins 2014b: 65), one becomes a contributory expert ‘by working with other contributory experts and picking up their skills and techniques’. Interactional expertise, on the other hand, can be acquired by deep immersion in the linguistic discourse of the relevant scientific domain.

The distinction between interactional and contributory expertise makes sense if one wants to theorize the role of a scientific expert, more precisely, the role of an experimental scientist, and Collins’s own work has nearly exclusively focused on such cases. The notion of a core-set of scientists, which he introduced in his study of gravitational wave research (Collins 1985), has later been applied in his work about expertise for policy decisions (Collins and Evans 2002). What counts for him are core-set competencies that can only be acquired through practical participation on the laboratory bench. Only in this way can scientists gain the important tacit knowledge, and only in this way can scientists as experts claim to make competent statements.

The laboratory as the site of knowledge creation, and the scientific institute which signals competence of the researcher and thus makes her a ‘certified expert’ is not the only source of expert knowledge, and it is arguably not the most important one when it comes to political decision-making (see Jasanoff 2003 for a similar critique).^9^ Laboratories as sources of power have been posited by several authors conducting ethnographies of laboratories in the 1970s and 80s (Latour 1983). This special focus has perhaps led to an unwarranted generalization in terms of political theory.

Another aspect of the core-set terminology is worth noting. In his book *Changing Order*, Collins drew attention to the fact that core-sets are not only or mainly distinguished by competencies but by gate-keeping activities. Collins argued that social factors crucially decided what counted as expertise, because there is no independent criterion available that could provide a standard for judging the competence of researchers and their knowledge claims at the frontier of new knowledge creation. Remember: *Changing Order* dealt with the problem of experimental replication and the attendant issue of experimenter’s regress. In Collins’s later work on expertise, the contributory quality of core-sets is contrasted with the interactive expertise of those outside the core-set. The separation is made on the basis of core competence, not on the basis of social mechanisms of gate-keeping. In *Changing Order* Collins was at pains to show that there was nothing inherently superior in one researcher’s quality or experimental skill compared to another. All depended on the social processes of gaining a standing within the core set. Now he holds that the sociological observer can evaluate the relative merit of expertise. This is a radical shift in perspective and not mentioned prominently.^10^ Hand in hand with the switch in perspective goes the adoption of a ‘realist’ theory of expertise, such that an expert remains an expert even if no clients acknowledge their expertise. This ‘realist’ theory of expertise is opposed to the ‘relational’ view of expertise that I put forward in this paper.

Bruno Latour, like Collins, started his academic career doing laboratory studies and, like Collins, conceives of laboratories as sources of power. He does not deal with the role of the expert in society in a separate publication. In his oeuvre he mentions the notion of expertise in passing, referring to the meaning of a specialist with experience, quoting the term *experitus*. For him an expert seems to be a specialist who has the luxury to work in the dark, where he can commit many errors, in contrast to the politician, who has to make all errors in public (Latour 1993: 225).^11^ Latour’s real attention is less on the role of experts, but on the role of science and scientists, on the role of laboratories as sources of political power, and on the similarity between science and politics.

His framework of actor-networks dispenses with explanation and only tries to describe networks, their reach, composition and growth over time. Through this description he wants to show, and critically examine, the genesis of established facts. However, established facts here pertain to a situation where despite uncertainty, and a competition of knowledge claims, a society comes to a conclusion of how to regulate a specific problem. This is why Latourian networks have no limits, and it does not make sense to speak about ‘natural’, ‘social’, ‘political’, or ‘scientific’ factors. This flat ontology does not recognize the problem of political decision-making as a discrete issue, and the role of expertise in the process.^12^

This short review of laboratory studies indicates that they are a mixed blessing for the ability of the field of STS when tackling the problem of expertise in society. They have focused on scientists and their practices, and their involvement in a wider net of relations. Political and institutional analysis has been a weak point in this field. In what follows I will look at contributions from authors that have a different theoretical agenda, focusing on the question of political decision-making in modern democracies where expert knowledge is seen as vitally important.

### Expertise and Counter-Expertise: The Politics of Knowledge

Back in the 1980s, analysing controversies about leaded gasoline, IQ testing, and smoking and lung cancer, Collingridge and Reeve (1986) distinguished between two scenarios where specialist knowledge and political decision-making are linked. In the first (‘under-critical model’) a policy consensus exists before research is undertaken. Scientific evidence merely legitimizes predefined policy options. In the second (‘over-critical model’) there is a succession of claims from experts and counter-experts without any agreement. Instead of a policy consensus we get endless technical debates. Collingridge and Reeve contrast several myths and realities of science and decision-making, for example, that science yields true and reliable knowledge (which they think is a myth), whereas in reality politicians use scientific information to justify their decisions. This leads them to abandon the idea that expertise is something that can be derived from the model of scientific research. Quite rightly they point to the fact that decision-makers are used to decisions under uncertainty, they do not try to collect comprehensive data before making a decision (see also Lindblom 1959).^13^ Nevertheless, reference to scientific knowledge claims seems to be important because all lobby groups in a policy arena tend to use them, and because scientific knowledge has higher prestige than other forms of knowledge.

‘The role of scientific research and analysis is therefore not the heroic one of providing truths by which policy may be guided, but the ironic one of preventing policy being formulated around some technical conclusions. Research on one hypothesis ought to cancel out research on others, enabling policy to be made which is insensitive to all scientific conjectures’ (Collingridge and Reeve 1986: 32).^14^

However, their distinction between two modes of decision-making (under- and over-critical) seems too rigid to cover the dynamics of the politics of knowledge. After all, sometimes we do get policy decisions after a period of seemingly endless technical debate. And there are examples where a policy consensus is undermined by emerging knowledge claims, as witnessed by constant scientific and technological innovation. While the authors make an important point that in modern societies knowledge is likely to be used in legitimizing or blocking specific political decisions, their notion of expertise is largely restricted to scientific expertise.

### Regulatory Science

In their study of advisory committees Salter et al. (1988) argued that there is a significant difference between science and scientific research, on the one hand, and knowledge that is applied to solve policy issues, on the other. They called the latter mandated science, in order to draw attention to this type of science that is not the outcome of an autonomous research process (à la Merton or Polanyi), but commissioned by public agencies keen to get specific and practical advice on regulatory policy issues.

‘Our image of scientists pictures them at work in the laboratory; we seldom raise the question of how scientific information moves from the laboratory to the world of politics and policy making’ (Salter et al. 1988: 1).

The work of such committees uses all the terms we are used to hear about scientific research, such as literature review, or peer review, but the purpose of mandated science is not to produce new scientific findings. Its point is to make a judgement about multiple sources of evidence, resulting in a recommendation to a pressing problem of public policy. The authors put it this way:

‘Mandated science must be understood as a separate sphere of scientific work… Increasingly, decision makers and their publics are placed in a quandary. On one hand they are increasingly dependent on science and scientists… on the other hand it is increasingly apparent that science cannot provide the clear answers that governments seek, at least not at the time when regulatory decisions are required. Moreover, science often provides conflicting answers …’ (Salter et al. 1988: 4).^15^

While the book by Salter at al. received little attention, Sheila Jasanoff’s work on advisory committees was to become highly visible, mainly through her book *The Fifth Branch* in which she addresses the issue of expertise, using the term regulatory science. In the opening chapter she lists three major findings from the sociology of science ‘that must be taken into account in any serious discussion of scientific advising’ (Jasanoff 1990: 12). These are: (1) Scientific facts are socially constructed; (2) Scientific paradigms and social prestige are important for problems facing advisory committees; (3) Through boundary work scientists decide who belongs to relevant professional and policy communities, thus holding up an appearance of scientific authority even in the face of uncertainty. Like Salter et al., Jasanoff draws an explicit comparison between conventional science and regulatory science. In so doing she addresses a shortcoming of Collins’s work (mentioned above). Experts are not only or mainly in their position as experts because of their technical competence but because of mechanisms of social inclusion/exclusion.

Jasanoff contrasts a technocratic with a democratic model of science advice (see also Habermas 1971; Irwin 1995) and concludes that neither ‘captures accurately what is at stake in decisions that are at once scientific and political. The notion that the scientific component of decision-making can be separated from the political and entrusted to independent experts has effectively been dismantled by recent contributions of the political and social studies of science’. And, even more strongly: ‘The idea that scientists can speak truth to power in a value-free manner has emerged as a myth without correlates in reality’ (Jasanoff 1990: 17, echoing Collingridge and Reeve). But is this the most important question to ask when trying to understand the role of expertise in decision-making? Would the science policy world be different if scientists were providing advice in a value-free manner? I suggest that it is more relevant to emphasize that the qualification and skill of the scientists is not closely linked to the decision context. Their knowledge often cannot provide the answers decision-makers request, as Salter et al. have shown.

Outlining the structure and rationale of the argument of her book, Jasanoff emphasizes the central role of scholarship from the sociology of science, beyond the ‘expected’ fields of law, political science and policy analysis. While the central role of knowledge is highlighted, the focus on its scientific nature posits a problematic, and perhaps unwarranted, assumption.^16^

Writing on experts as policy advisors, Jasanoff (1990: 229) points out that ‘experts themselves seem at times painfully aware that what they are doing is not “science” in any ordinary sense, but a hybrid activity that combines elements of scientific evidence and reasoning with large doses of social and political judgment’. This statement captures perhaps the most important aspect of expertise. In a more recent paper, Jasanoff (2011: 21) emphasizes the role of the expert as a translator or mediator between knowledge and decision-making, quite akin to what I propose here. However, her concept of the expert seems to depict them as professionals: ‘it is not science per se that speaks unmediated to power, but […] the bridge between science and politics is built by experts, a cadre of knowledgeable professionals’. Occluded from this conceptualization is a kind of expertise that is not based on esoteric science, and not located within a profession. We shall now turn to a body of literature that conceptualizes the role of lay expertise.

### Lay Expertise

Brian Wynne (1996) is usually credited with the insight that lay people can be experts, too. His famous study of Cumbrian sheep showed farmers exposed to the radioactive fallout from the Chernobyl accident in 1986 and the government interference in their daily farming practices. Wynne argued that while government scientists made authoritative statements about the radioactive decay in the farmers’ soil, the farmers themselves knew something else about the nature of their herds and the requirements of animal husbandry. After all, the issue was how to solve the tension between radiation risks and maintaining the farmers’ livelihoods. However, the government scientists and the farmers had specialized knowledge about different things, and both had their own interests. The local knowledge of the farmers was based on experience and self-interest, while the government experts relied on abstract models and an interest to reassure the farmers and the public, apart from keeping their monopoly status as provider of certified, reliable knowledge.

Wynne’s call for an inclusion of the farmers in decision-making, and being critical of the arrogance of state authority has proven immensely influential with scholars in STS and deliberative democracy circles. But there is a question to be asked about which social groups represented which expertise. Wynne shows how government scientists provided laboratory and abstract expertise that first gave false reassurances but then ordered a drastic restriction in movement of sheep. It is not clear what difference in policy recommendations would have been following from the farmers’ lay expertise, apart from the obvious economic aspect of financial compensation for the loss of sheep.

Wynne does provide some hints about the economic dimensions of the decisions at stake. Hill farmers were dependent on their lambs, which were raised after Easter and sold in the autumn, mainly to European markets. They needed to be sold at the right time, being neither too lean, nor too fat. Any indication or suspicion that the lambs were radioactively contaminated would massively devalue them and thus pose an immediate and direct threat to the farmers’ livelihoods. What was the right thing to do given the circumstances of radioactive contamination of soil and herds? This is a question that is never explicitly addressed in Wynne’s account. We hear about the distrust of local farmers vis-à-vis the government scientists and a history of local radioactive pollution, following the Windscale disaster in 1957, which was probably covered up by government secrecy. But given the situation post-Chernobyl, what were the options for the farmers, and for the government? What was in the public interest, and what in the farmers’ interests? Was there a difference between the two? How did the expertise of the government scientists and that of the Cumbrian farmers answer these questions? While Wynne’s account tells the story of an apparently confused and haphazard government intervention it does not tell us much about the farmers’ demands with regard to managing the crisis.

For example, Wynne relates that ‘[a]lthough the farmers accepted the need for restrictions, they could not accept the experts’ apparent ignorance of the effects of their approach on the normally flexible and informal system of hill farm management. This experience of expert knowledge being out of touch with practical reality and thus of no validity was often repeated with diverse concrete illustrations in interviews’ (Wynne 1989: 34). In addition, conflicts about the rules for compensation emerged which left the farmers embittered.

The culture and social identity of the hill farmers are mentioned as important factors when assessing the divergent ways of dealing with the risk of radioactive fallout on the Cumbrian hills. As Wynne points out, the farmers’ expertise was not codified anywhere, it was passed down the generations orally and by apprenticeship.^17^

In sum then, Wynne’s point is not that the hill farmers have developed some kind of counter-expertise that would have led to different policy recommendations. Rather, he is concerned about the interests and social identity of the hill farming community. The only engagement the farmers had with government experts from the Ministry of Agriculture, Fisheries and Food highlighted its contradictory nature, leading to completely different courses of action.^18^

Michel Callon (1999) has taken up the point about the patronising effects government scientists can have on citizens. He emphasizes the complementarity of expert and lay expertise, acknowledging that both are prisoners of their own beliefs but that lay experts, in addition, fear ‘that that someone else may decide for them what is good for them, and that such decisions would be taken without the slightest knowledge of their needs or wishes’ (Callon 1999: 88; see also Roszak 1969).

This is an important insight. It underlines the fact that different forms of expertise may be competing, and that specific kinds of expertise align with specific social interests. Callon mentions those affected by official expertise, a scientific or technocratic undertaking that is perceived as threatening by those affected. One could argue that these scientific experts themselves are trying to protect their interests, too. But there is a difference in that the lay experts often cannot act in a symmetrical way, and thus they do not have the same kind of control over events.

Callon’s approach would be compatible with a view that defines expertise in the way I am proposing here. In this view, both lay experts and official experts mediate between a body of knowledge and decision contexts. Both need to fight for recognition and acceptance (albeit the official expert may have an advantage in this game, especially under conditions where access to elites is important). The nature of the knowledge in both cases is not essential to the framework: it could be a body of scientific knowledge, it could be a body of practical knowledge, of tacit, or secret knowledge (see Stehr and Grundmann 2005).^19^ The important aspect is that expertise mediates between a body of knowledge and its application. Scientists could fulfil this role but they are not the only group.

Wynne and Callon have touched upon the notion of stakeholder inclusion which becomes even more prominent in the framework of Post-normal Science, a situation in which ‘[s]takes are high, facts are uncertain, values in dispute, and decisions urgent’ (Funtowicz and Ravetz 1990; Ravetz 1999). This framework explicitly drops the distinction between the action-oriented urgency of politics and the absence of such pressure within science, a distinction introduced at the beginning of this paper and emphasized by Collins.

In postnormal situations science and decision-making become closely coupled. Does this bridge the gap between science and politics? Or is this entanglement a hindrance for decision-making? The authors believe that credible solutions must rely on the legitimation of public participation. Science should stop pretending that it can provide reliable knowledge for decisions. Under conditions of uncertainty and value conflict, experts and lay people are alike, and should be given equal treatment in the process:

‘Persons directly affected by an environmental problem will have a keener awareness of its symptoms, and a more pressing concern with the quality of official reassurances, than those in any other role. Thus they perform a function analogous to that of professional colleagues in the peer review or refereeing process in traditional science, which otherwise might not occur in these new contexts’ (Ravetz 1993: 649).

In a similar way to Wynne, the authors hold that official expertise and lay expertise should be seen as complimentary, not as antagonistic:

‘The Post-Normal Science approach should not be interpreted as an attack on the accredited experts, but rather as assistance. The world of “normal science” in which they were trained has its place in any scientific study of the environment, but it needs to be supplemented by awareness of the ‘post-normal’ nature of the problems we now confront’ (Ravetz 1993: 653).

Here the stakeholder engagement is modelled explicitly on the institution of peer review as practiced within scientific communities. But it is not obvious what the reason for inclusion is: is it the lay knowledge of stakeholders, or is it the democratic principle of inclusion of stakeholders, irrespective of their status as experts?

If lay people have special knowledge that is thought to be essential, or at least an important complement to the specialist (scientific or professional) knowledge, this needs to be spelled out. If, on the other hand, lay people are seen as equally entitled to make judgements in the face of uncertainty, as the experts are, then the rationale for including them is a principle of democracy.

Yearley (2000: 109) states: ‘Funtowicz and Ravetz stress that citizen involvement is proposed by them not because they are committed to the furthest extension of democracy, but because the involvement of a larger group of peers, with different kinds of knowledge, will be beneficial to the production of high-quality knowledge’. In this interpretation PNS would improve our knowledge of the world, where in another reading it would lead to better decisions. Proponents of this latter view have pointed out that there is a similar interest in policy studies which should be taken into account:

‘It is crucial now for scholars of PNS to make explicit reference to other heuristic concepts such as ‘‘deliberative policy analysis’’ (…) which emphasize the importance of governance and institutionalizing of participation and account for social construction in politics and science’ (Turnpenny et al. 2011).

In contrast to science-based notions of expertise, the contributions in this section emphasize lay expertise and lay-expert interaction and thematize the importance of stakeholder participation. However, they stop short of conceptualizing the fundamental difference between knowledge production and decision contexts, and the attendant question of the specific properties of the knowledge, ‘the type of knowledge’ that can be used for decision purposes. It does not address in sufficient detail how stakeholder representation relates to the question of knowledge production and application.

The upshot of this section is that expertise is conceptualized in largely scientific terms, or else tries to involve lay people in decision-making where it remains unclear what their expertise rests upon. Expertise is discussed either in close analogy to a scientific ideal, neglecting the role of non-scientific experts (field experts), or ideals of political participation are brought in which are modelled on scientific practices (extended peer review). Also unexamined is the relation between interests and ideas which seems to be crucial in this context.

### Honest Brokers

A conceptual refinement of the role of the scientist as expert in the policy advisory process has been developed by Roger Pielke Jr. (2007). His typology identifies different roles of scientists engaged in different ways with a decision-making process. These roles are presented as pure scientist, science arbiter, issue advocate, and honest broker. He calls them experts, but on several occasions he also uses the term ‘scientists and other experts’ (without defining what these ‘other experts’ might be). The pure scientist has no interest in the decision-making process and simply wants to share information about facts. ‘The Science Arbiter serves as a resource for the decision-maker, standing ready to answer factual questions that the decision-maker thinks are relevant. The Science Arbiter does not tell the decision-maker what he or she ought to prefer’ (Pielke Jr. 2007: 2).

In contrast, the issue advocate tries to convince the decision-maker of one best course of action. Finally, the honest broker leaves it to the decision-maker to reduce the options and to make a choice: ‘The defining characteristic of the honest broker of policy alternatives is an effort to expand (or at least clarify) the scope of choice for decision-making in a way that allows for the decision-maker to reduce choice based on his or her own preferences and values’ (Pielke Jr. 2007: 2–3).

A characteristic of both honest brokers and issue advocates is an explicit engagement of decision alternatives whereas the pure scientist and science arbiter are not concerned with a specific decision, but instead serve as information resources.

Unlike the science arbiter, the honest broker seeks explicitly to integrate scientific knowledge with stakeholder concerns in the form of alternative possible courses of action.

I noted above that scientists often do not have the knowledge which could serve and justify a specific policy. This gap between what is known in scientific terms and what would be needed to know for practical purposes can be exploited on both sides of the science policy interface. Pielke Jr. (2007: 77) argues that:

‘Contemporary science policies create strong incentives for scientists to wage political battles through science by emphasizing the roles of Pure Scientist and Science Arbiter in all cases, when in fact an advocacy position is actually being expressed. At the core of the argument presented here is a critique of the longstanding expectation that science can and should be separated from considerations of the applications of science’.

Pielke argues that the social influence of experts on the policy process is strong:

‘Democracy is a competitive system in which the public is allowed to participate by voicing their views on alternatives presented to them in the political process. Such alternatives do not come up from the grassroots any more than you or I telling an auto mechanic what the options are for fixing a broken car. Policy alternatives come from experts. It is the role of experts in such a system to clarify the implications of their knowledge for action and to provide such implications in the form of policy alternatives to decision-makers who can then decide among different possible courses of action’ (Pielke Jr. 2007: 12; see Brown 2008 for a critique).

Two aspects need further elaboration here; for one, it is not clear that only experts (in the sense of scientific experts) introduce policy options into the political process. Like previous frameworks reviewed here, Pielke conceives of experts by and large as scientists of some sort. Secondly, the notion of honest brokers could be seen as misleading. It could indicate that other roles are not honest, or that some brokering is not honest. It would be a difficult judgement, depending on the merits of each case, to evaluate the honesty of such brokering. The main problem with the term, however, is the suggestion that experts as experts could somehow be independent from the decision process which they have been asked to join. Brokers make matches, select options, and suggest courses of action. In this sense experts are brokers^20^, and it would be problematic to restrict their role to a widening of policy options. Perhaps Pielke does not see this because he holds on to the ideal of impartiality as a virtue of experts.^21^

## Discussion

The argument so far has explored different conceptualizations of experts and expertise. It has advocated a relational concept of expertise, and emphasized the proliferation of expertise in the knowledge society. Several (but not all) of the approaches discussed above focus on scientists as providers of expertise, and all thematize government and private citizens as clients of expertise. The role of expertise for business and NGOs seems to be a blind spot.^22^

This still leaves open the question of how the expertise relates to decision-making.

Let us revisit Turner’s work and an intriguing comment on expertise provided to a public authority. He says the problem with advisory bodies is that ‘the evidence is not enough to guide practice unequivocally, and that *some sort of fraught step, even a leap, is needed to get from the evidence to practice*. The reason for these institutions is this: the science alone—the ‘‘evidence,’’ in this phrase—is insufficient, conflicting, or simply not in a form that can guide practice. But practice is necessary, ongoing, and consequential. So the process of bridging the gap needs to be helped along’ (Turner 2010: 253, orig. emph.).

This very insight brings us back to the beginning of this paper and the sociological notion that knowledge and action are often disconnected and that action often precedes knowledge. The example of advisory bodies advising high politics is instructive here, and also for other types of expertise. Robert Oppenheimer famously suggested that ‘there are formidable differences between the problems of science and those of practice. The method of science cannot be directly adapted to the solution of problems in politics and in man’s spiritual life’ (cited in Shapin 2008: 70).

A private person, like a government, knows that the evidence often is not enough—it may be contradictory or obscure. A leap is necessary, which all too often is a leap of faith. An advisor can bridge the gap, so to speak. The same logic applies to other social groups requesting expertise, such as companies and civil society originations. If this is the case, the social attributes of expertise come to the fore. They have been described as defining an issue, reducing complexity, creating trust, and identifying courses of action (Stehr and Grundmann 2011). Nevertheless, there is a difference between citizens searching for expertise, and corporate bodies doing so. The former is usually genuinely interested in the best advice for the best course of action, while the latter has either chosen a decision for which expertise can provide legitimation, or else wants to use expertise in a political power game.

The link between knowledge and decision-making has been obscured or neglected in the literature to some extent. One can speculate about the reasons. It could be that several approaches after the 1970s (post ‘wave 1’ in Collins’ terms) have given up on a conceptual separation between science and politics (but see Luhmann 1990; Merton 1973; Weingart 2001). The resulting conceptual ‘hybrid’ frameworks have led to three strategies in dealing with the problem of expertise:

1. Equating expertise with scientific expertise while at the same time conceptualizing scientific expertise as a hybrid of science and politics (and possibly more, as expressed in the frameworks of ‘co-production’ and Actor-Network-Theory);
2. Equating theoretical knowledge with practical knowledge;
3. Elevating (or diminishing) lay expertise without making sufficiently clear what epistemic status lay knowledge has.

In so doing, a potential confusion has arisen that makes it difficult to investigate the link between knowledge and decision-making. In strategy 1 such a link would be an artifact of an obsolete distinction between science (or more generally: knowledge) and politics. This verdict is borne out of many studies in STS that have shown that in specific cases of science policy interactions it is impossible to demarcate science and politics. Maybe such a claim is the result of a specific research interest (historical reconstruction) and research methodology (case studies). A research programme of ‘following the actors’ (Latour 1987) will show persons and institutions that are embodiments of several roles and functions. For such an approach posing the theoretical question of how knowledge and decision-making are linked becomes an anomalous question that cannot be answered—or has been answered in that the link is always already present.^23^

In strategy 2 little attention is paid to the question what kind of knowledge is needed for decision-making. More could be said about this, making use of the distinction between knowledge for practice and practical knowledge (see Grundmann and Stehr 2012 for an extensive discussion). This needs emphasizing, since the pragmatic relevance of knowledge cannot be assumed *a priori*. Several conditions must be met if knowledge is to be turned into knowledge for action. The successful application of knowledge in concrete situations for action requires that knowledge is geared towards application (often it is not); that possibilities for action exist; and that the decision-maker has appropriate latitude for action, i.e. is able to shape events. These different elements must be linked together, in order that knowledge may become practical knowledge through decisions and their implementation. In other words, scientific knowledge is not sufficient–other knowledge forms may well be required–and experts needs to be able to identify levers of action that exist in reality and that can be moved (Stehr and Grundmann 2011).

The insistence on the difference between esoteric scientific or professional knowledge and lay reasoning is valid but misguided. Of course, there are differences in skill and mastery of a specific domain of knowledge. What is relevant for decision contexts is the ability to relate knowledge claims to the decision-making context, to define a situation and to identify options for intervention. Often there is competition in the supply of these services, but not always, as the case of law seems to show. If one were to privilege scientists and professionals, this would not necessarily lead to better advice (as their esoteric knowledge could be irrelevant or detrimental for action) but put them into this role *qua authority*. Knowledge without expertise is the specter haunting this approach.

In strategy 3 the fact is recognized that expertise is widely distributed in society and that expertise is not to be assimilated, or restricted to scientific knowledge. Nevertheless, the problem remains of how one can understand this kind of expertise in terms of its epistemic contribution to decision-making. Collins’s term ‘interactional expertise’ defines this as a great achievement but sees it still lacking the final ingredient to be complete, ‘contributory’ expertise. Wynne’s term lay expertise seems to indicate that there are ‘real’ experts, and then there are lay experts, who happen to have an interest in a social or political issue. The term stakeholder normally describes this role, and not all stakeholders are *per se* experts. They may articulate an interest in an issue, preferring one decision to another, based on their special interests. It is an open question  of how they contribute to the understanding of an issue and what kind of expertise they contribute. In principle, lobby groups can make valid points, even if they are surrounded by suspicion (‘they would say that, wouldn't they?') Be that as it may, stakeholders as lay experts cannot be expected to be impartial (which is not to say that conventional experts, traditionally conceived, are impartial either).

## Conclusion

The notion of expertise in knowledge societies poses a theoretical challenge. Several approaches in STS (with some notable exceptions) have used the ideal of the scientist when conceptualising expertise, and conceive of expertise as something to be possessed. This neglects the relational aspect of expertise. The rise of field experts is acknowledged by many authors but their role as stakeholders and interest groups vis-à-vis knowledge producers remains obscure.

The review of five frameworks on expertise has revealed some interesting features, and some lack of perspective. The interesting features have to do with the limited function of science to provide reliable knowledge for practical political purposes, and a need to complement the decision-making process with stakeholder groups that originate outside science. There is a growing realization that expertise is ubiquitous. However, this literature also shows to some extent the fixation with scientific and professional knowledge as the standard of reference when talking about expertise, and a neglect of political and institutional aspects of decision-making (see also Jasanoff 2003).

Future research needs to take into account relevant contributions from political science, policy studies, the sociology of professions, and international relations (Abbott 1988; Eyal 2013; Fischer 2009; Haas 2004; Jenkins-Smith and Sabatier 2008). The literature on policy networks seems especially relevant in this context (Grundmann 2001; Moran et al. 2008). There are lessons to be taken on board across disciplines. However, for the present purpose, the point is to demonstrate the benefit of a conceptual innovation that understands expertise as relational, and as mediating between knowledge production and application, thus enabling novel ways of dealing with expertise in the knowledge society, linking knowledge to decision-making.

## Footnotes

1 In addition, there are ‘moral experts’, such as a Kantian philosopher or a Catholic theologian, who are experts but not impartial. Thanks to Mark Brown for this observation.

2 The notion of knowledge broker comes close to the problem but, at least in the definition of Meyer (2010) is too focused on the cognitive aspects, and the translation of knowledge to audiences, leaving aside the aspects of decision, advice and action. Meyer (2010) writes: ‘[B]rokering involves a range of different practices: the identification and localization of knowledge, the redistribution and dissemination of knowledge, and the rescaling and transformation of this knowledge. Brokering knowledge thus means far more than simply moving knowledge—it also means transforming knowledge’.

3 See Kantrowitz (1975) for a discussion of this problem and the suggestion of an experimental institution of a ‘science court’.

4 In 2012, 41% of Americans expressed “a great deal of confidence” in leaders of the scientific community; this proportion has stayed nearly stable over the last 40 years. Compared to other social domains they do well (NSF 2014). Eurobarometer (2010) does not have directly comparable data. Asked about controversial scientific and technological issues Europeans feel that scientists cannot be trusted to tell the truth because they depend more and more on money from industry. More than half (58%) agrees that this is the situation and only 16% disagree.

5 Collins (2014b) traces the suspicion of expertise to the 1960s counterculture and its legacy in today’s social science scholarly community. While this captures one aspect of social context, it does not address the deeper nature of social change that has led to a proliferation of knowledge sources. In other words, the criticism of elite expertise (and the demand for ‘more democracy’) is not only a feature of social science scholarship but also a social reality.

6 Reasons for this rising demand have been identified as a loss of tradition, rise of individualism, complexity of social life, the growth in the range of social actions in which individuals engage and the attendant insecurity (see Beck 1992).

7 In this process experts often disagree, as (Mazur 1973: 251) noted: ‘The theories, models, procedures and formulae of science and technology are generally believed to allow one trained in their use simply to calculate an unambiguously correct answer. A technologist or scientist soon comes to recognise that the complex technical problems of the state-of-the-art require subtle perceptions of the sort which cannot be easily articulated in explicit form. When it is necessary to make a simplifying assumption, and many are reasonable, which simplifying assumption should be made? When data are lacking on a question, how far may one reasonably extrapolate from data of other sources? How trustworthy is a set of empirical observations? These questions all require judgements for which there are no formalised guides and it is here that experts frequently disagree’.

8 The notion of lay expertise is mainly applied in research about medial settings, see, for example, Akrich and Rabeharisoa (2012). The *locus classicus* is Epstein (1995: 408) who claims that ‘AIDS treatment activists have constituted themselves as credible participants in the process of knowledge construction, thereby bringing about changes in the epistemic practices of biomedical research’. It is beyond the scope of this paper to assess to what extent this is a valid description or if this is another instance of emerging expertise at the interface between knowledge production and application.

9 Besides, one would expect that deep immersion for an extended period of time would allow participants to develop contributory expertise. It is surprising that Collins thinks otherwise. Maybe he overgeneralizes his own experience when he says that decades of close contact to the community of gravitational wave researchers has not allowed him to become a contributor, even after he successfully accomplished something like a Turing test (Collins 2014b: 69–71).

10 Collins might reply that he provides the rationale of the ‘three waves of science studies’ to justify this re-orientation. *Changing Order* would be a testimony to his working within wave two which he now thinks to be limited, and in need of extension.

11 Perhaps this was written with the example of France in mind. In the USA, being an expert does not protect them from being publicly exposed and even sued.

12 However, in a recent talk about climate change, Latour (2013) does consider the nature of politics which he defines as an agonistic field, following Carl Schmitt. Perhaps the turn from ‘matters of fact’ to ‘matters of concern’ (see Latour 2004) has led him to consider a framework in which political aspects become important and can be addressed as such. In his book *Politics of Nature*, Latour also outlines a set of different institutions with specific functions (see Brown 2009).

13 The European climate change policy development is a case in point. It is perhaps little known that at the time of the founding of the Intergovernmental Panel on Climate Change (IPCC), EU officials declared that we know enough in order to act (Liberatore 1994: 192).

14 This is similar to Max Weber’s statement that ‘the ruler…keeps one expert in check by others’ (Weber 1978: 995).

15 See also (Weinberg 1972: 209) who famously said that there are ‘questions which can be asked of science yet cannot be answered by science’.

16 Jasanoff’s arguments about expertise are informed by the choice of her cases, which are scientific and professional advisors providing expertise for government, see Table 1.

17 It could be suggested that the hill farmers relied on a highly complex and tightly coupled system which made failure under stress likely, cf. Perrow (1999).

18 There seems to be some evidence that the hill farmers had a better relationship with the local Institute for Terrestrial Ecology which ‘was most fortunate in this respect because, as a locally based institution, it had the closest such practical contact. Through the farmers’ informal grapevine, it subsequently gained a reputation as being plainspeaking, open about uncertainty, independent, and trustworthy’ (Wynne 1989: 38).

19 The asymmetry between corporate and government expertise, on the one hand, and lay expertise, on the other, is perhaps most pronounced in the area of surveillance, as Edward Snowden’s revelations have shown.

20 This goes against the definition given by Meyer cited above.

21 As has been noted, honest brokering in Pielke’s sense is most likely to apply in collective advisory bodies, and not at the level of the individual expert (see Grundmann 2012).

22 For an exception with regard to business expertise, see Pollock and Williams (2016). Of course, business consultancy is a constant topic in the management literature but does not advance theoretical frameworks.

23 Of course, there is also much insightful work in STS studies of expertise that shows that the separation of science and politics is a practical result of boundary work (Gieryn 1983; Jasanoff 1990). In this way scientists are presumed to have agency in deciding what exactly counts as legitimate scientific knowledge and practice, and what does not.
